# Biopolymer‐Based Food Packaging: Functional Properties, Enhancement Strategies, and Future Perspectives

**DOI:** 10.1002/fsn3.72005

**Published:** 2026-06-07

**Authors:** Songul Bayrak, Mehmet Akif Omeroglu

**Affiliations:** ^1^ Department of Chemistry, Faculty of Science Ataturk University Erzurum Turkey; ^2^ Department of Molecular Biology and Genetics, Faculty of Science Ataturk University Erzurum Turkey

**Keywords:** active packaging, barrier properties, bio‐nanocomposites, circular economy, edible coatings

## Abstract

The increasing global demand for food and growing environmental concerns have intensified efforts to develop sustainable alternatives to conventional petroleum‐based food packaging materials. Food packaging plays a critical role in preserving product quality, ensuring food safety, and extending shelf life across the supply chain. However, the extensive use of non‐biodegradable plastics has contributed significantly to environmental pollution and resource depletion, encouraging the development of renewable and eco‐friendly materials. Biopolymers have attracted considerable attention as greener alternatives due to their biodegradability, reduced carbon footprint, and the ability of optimized formulations to achieve oxygen barrier properties comparable to certain petrochemical plastics. This review summarizes the sources, classifications, and key functional properties of biopolymer‐based packaging materials including barrier, mechanical, optical, and thermal characteristics. Recent advances in active packaging systems such as antimicrobial and antioxidant biopolymer‐based materials are also discussed. Nevertheless, despite these advantages, major limitations, including high moisture sensitivity, weak mechanical performance, and limited scalability, still hinder their widespread industrial application. In addition, substantial research gaps remain regarding long‐term performance and real‐world environmental impact. Applications in different food sectors are examined alongside environmental and regulatory aspects. This review provides a critical evaluation of performance enhancement strategies, including polymer modification, nanocomposites, and bioactive incorporation, highlighting both their effectiveness and limitations. Compared with conventional plastics, biopolymers generally exhibit lower barrier and mechanical properties, yet recent studies indicate that nanocomposite approaches can improve barrier performance by up to 30%–50%. Overall, biopolymer‐based packaging represents a promising approach for sustainable food systems. Future research should focus on bridging the gap between laboratory‐scale innovation and industrial implementation, particularly in terms of cost‐efficiency and large‐scale production.

## Graphical abstract

Biopolymer‐based food packaging offers a sustainable alternative to conventional petroleum‐derived plastics. Functional enhancement through nanocomposites, active packaging systems, and polymer modification significantly improves barrier performance, mechanical strength, and food preservation. These developments contribute to reduced environmental impact while addressing key challenges in large‐scale industrial implementation.

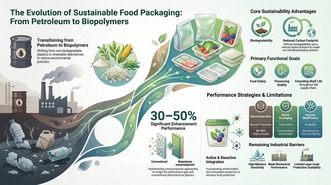

## 1 Introduction

With the continuous growth of the global population, the demand for food is also increasing. Recent estimates indicate that over 400 million tons of plastic waste are generated annually worldwide, of which nearly 40% is attributed to packaging applications. Notably, a large fraction of this waste is derived from single‐use food packaging, highlighting the urgent need for sustainable alternatives (Organisation for Economic Co‐operation and Development 2022). Reducing food losses can help alleviate malnutrition and contribute to meeting future global food demands. Therefore, minimizing food loss and waste is essential to ensure global food security (Bajzelj et al. 2020). Among the most effective approaches for reducing food loss and waste are proper food preservation and packaging. Packaging plays an important role in modern food systems by facilitating the safe distribution and storage of food products. Its main purposes are to ensure food safety, maintain product quality, and improve storage stability by protecting foods from environmental, microbial, chemical, and physical hazards during storage and transportation. Despite fulfilling essential protective functions, conventional packaging materials raise critical environmental concerns, whereas biopolymer‐based alternatives still face challenges such as poor moisture resistance and limited mechanical strength. Bridging this performance gap is therefore essential for the development of green packaging systems. In addition to providing physical protection, packaging also serves as an effective barrier and offers the mechanical and optical properties necessary to maintain product quality and safety, thereby ensuring an acceptable shelf life (Mahmoudi et al. 2025; Verghese et al. 2015).

Consequently, nearly all food products are packaged at least once during the journey from farm to consumer. The characteristics and quality of a food product are strongly influenced by the type of packaging used, including its structure, design, and the materials from which it is made. Common packaging materials include paper, glass, plastic, metal, different types of cardboard, and composite materials consisting of more than one component such as plastic‐coated cardboard (Cheng et al. 2024; Kumari et al. 2024; Oladzadabbasabadi, Ghasemlou, et al. 2026). However, food packaging faces significant challenges related to environmental sustainability, production processes, consumer expectations, regulatory requirements, and the complexity of maintaining food safety while addressing environmental concerns. For this reason, it is essential to carefully evaluate the properties of packaging materials to develop systems that effectively fulfill their intended functions. These properties include barrier capabilities against gases (such as water vapor, oxygen, and carbon dioxide), aroma retention, light protection, fat resistance, migration potential, as well as physical and mechanical strength and hygienic characteristics, all of which are largely determined by the inherent nature of the material (Dilkes‐Hoffman et al. 2018; Ncube et al. 2021).

Conventional petroleum‐based plastics are widely used in food packaging because of their low cost, lightweight structure, flexibility, and strong barrier and mechanical properties. Common examples include polyethylene, polypropylene, and polyethylene terephthalate. Conversely, their non‐biodegradable nature and dependence on fossil resources have raised major environmental concerns including plastic accumulation, microplastic pollution, and greenhouse gas emissions. These challenges have accelerated the search for eco‐friendly packaging alternatives derived from renewable resources (Ncube et al. 2021). Moreover, plastic production and disposal contribute significantly to global greenhouse gas emissions (Geyer et al. 2017; Zheng and Suh 2019). Recent estimates indicate that plastic production accounts for approximately 3%–4% of global greenhouse gas emissions, with projections suggesting a notable increase by 2050 if current consumption patterns continue. In response, numerous policy‐driven initiatives, including bans on single‐use plastics and the implementation of circular economy strategies, have been introduced, particularly within the European Union and other developed regions, to reduce plastic waste and promote sustainable alternatives (Geyer et al. 2017; Kasznik and Łapniewska 2023). These environmental challenges have increased interest in circular material management strategies, particularly those based on circular economy principles aimed at reducing waste generation and improving resource efficiency.

The concept of a circular economy highlights the importance of the 4R principles, which are reduce, reuse, recycle, and recover, emphasizing that the generation and utilization of resources should prioritize sustainable practices when they are more environmentally beneficial than conventional petrochemical plastics (Geissdoerfer et al. 2017). In this context, biopolymer‐based packaging materials offer considerable opportunities for integration into circular economy systems. For instance, bio‐based and biodegradable polymers support the reduce and recover principles by minimizing dependence on fossil resources and enabling organic recycling through composting. Similarly, the development of reusable biopolymer films and coatings contributes to the reuse strategy, whereas the design of recyclable bio‐based composites aligns with the recycle principle. These approaches highlight the potential of biopolymers to bridge material performance with circular economy objectives, whose primary functions are to ensure food safety (Kakadellis and Harris 2020; Rosenboom et al. 2022). Accordingly, increasing research efforts have focused on the development of biopolymer‐based materials that can combine environmental sustainability with the functional requirements of modern food packaging systems. Consequently, a major challenge for society is to reduce the reliance on persistent plastic materials, whereas acknowledging that glass and metals, despite being non‐biodegradable, exhibit high recyclability and lower environmental leakage compared to single‐use plastics. Ongoing research studies are therefore focused on identifying environmentally friendly materials with suitable packaging properties that can reduce waste while supporting more sustainable packaging systems. Although numerous recent reviews have highlighted the potential of biopolymer‐based materials for sustainable food packaging (Flórez et al. 2023; Perera et al. 2023; Sharma et al. 2025), the present review offers a more comprehensive and critically integrated perspective by systematically linking material characteristics, functional performance, modification strategies, environmental sustainability, and industrial applicability within a single framework. In particular, this review emphasizes the relationships between structural characteristics and functional performance including barrier, mechanical, optical, thermal, and chemical properties of biopolymer‐based systems. Moreover, recent advances in polymer modification, nanocomposite technologies, crosslinking approaches, and bioactive incorporation are critically evaluated together with their associated limitations, safety concerns, and scalability challenges. The present work also highlights the gap between laboratory‐scale innovations and practical industrial implementation, whereas also addressing regulatory considerations and future commercialization perspectives for advanced biopolymer‐based food packaging systems.

## 2 Biopolymer‐Based Materials as Sustainable Alternatives to Conventional Food Packaging

The increasing environmental concerns regarding plastic materials have encouraged increased research studies into alternative materials for food packaging. Biopolymers have gained increasing attention as renewable and eco‐friendly materials derived from agricultural resources or biomass feedstocks, particularly due to their lower environmental impact and potential to partially replace conventional plastics in specific packaging applications. These materials can be readily degraded by wild‐type microbial strains under favorable environmental conditions including suitable temperature, oxygen levels, moisture, and soil environments. In addition to their biodegradability, certain biopolymers, particularly polyhydroxyalkanoates (PHA) and polylactic acid (PLA)‐based materials, can exhibit oxygen barrier properties comparable to conventional plastics such as polyethylene terephthalate (PET) and polypropylene (PP) under optimized processing conditions. For example, PHA films have been reported to exhibit oxygen permeability values lower than those of polyethylene (PE), whereas certain PLA‐based composites demonstrate tensile strength values approaching those of polypropylene (PP) (Koller 2022; Perera et al. 2023). Moreover, they are generally as considered biocompatible, renewable, safe, and environmentally friendly (Florez et al. 2022; González‐López et al. 2023). However, it is essential to note that the biodegradability of biopolymers is highly dependent on environmental conditions. In many cases, efficient degradation requires controlled industrial composting environments with specific temperature, humidity, and microbial activity, whereas degradation under natural environmental conditions may be notably slower or incomplete (Peelman et al. 2013; Rosenboom et al. 2022). Their production processes are also environmentally friendly and typically do not generate harmful residues. For these reasons, biopolymers are regarded as some of the most promising materials for food packaging applications (Peelman et al. 2013). Despite these advantages, they often exhibit certain limitations compared with traditional petroleum‐based plastics. These limitations include relatively poor barrier performance, weaker mechanical strength, limited processability, and higher production costs. These limitations are primarily associated with the intrinsic structural characteristics of biopolymers. For instance, the hydrophilic nature of many polysaccharide and protein‐based biopolymers leads to high water vapor transmission rates (WVTR), thereby reducing their effectiveness as moisture barriers. In addition, relatively weak intermolecular interactions and low crystallinity in certain biopolymer matrices contribute to inferior mechanical strength compared to conventional plastics. Furthermore, their sensitivity to thermal and processing conditions can result in structural instability during large‐scale manufacturing (He et al. 2025). The relatively high gas and water vapor permeability, brittleness, low thermal deformation resistance, and limited tolerance to prolonged processing conditions of many biopolymers restrict their industrial application (Abdullah et al. 2022).

To address these drawbacks, several strategies have been developed to improve the performance of biopolymer‐based packaging materials. One approach involves modifying the chemical structure of polymers, for example through block copolymerization, which enables the formation of polymers composed of different segments or through post‐polymerization modification of functional groups along the polymer backbone (Arrieta et al. 2013). Other strategies include the incorporation of additives such as plasticizers, antioxidants, antimicrobial agents, and nanomaterials, as well as blending biopolymers with other polymeric materials. Recent studies have demonstrated that lignin‐based nanocomposites can substantially improve multifunctional performance by improving UV resistance, antioxidant activity, and barrier properties, although challenges related to dispersion and scalability remain (Oladzadabbasabadi et al. 2025). These improvement strategies boost material performance through several underlying mechanisms. For example, the incorporation of nanofillers such as nanocellulose or metal oxide nanoparticles can create a tortuous diffusion pathway, thereby reducing gas and moisture permeability. Similarly, polymer blending and copolymerization can improve intermolecular interactions, leading to enhanced mechanical strength and structural integrity. The addition of plasticizers increases chain mobility, improving flexibility but potentially reducing barrier performance. Furthermore, bioactive additives such as antimicrobial and antioxidant agents provide functional activity by inhibiting microbial growth and oxidative degradation, thereby extending the shelf life of packaged foods (Arrieta et al. 2013; He et al. 2025). Additionally, the incorporation of natural or synthetic nanofillers can enhance the physical and mechanical properties of these materials. By combining polymer matrices with fillers obtained from renewable resources, fully compostable nanocomposites can be developed for sustainable packaging applications (Rhim et al. 2013). Although nanofillers considerably increase the functional properties of biopolymer‐based packaging materials, their potential safety risks must also be thoroughly addressed. One of the primary concerns is the possible migration of nanoparticles from packaging materials into food, especially under conditions such as elevated temperatures, extended storage periods, or contact with acidic foods. The extent of nanoparticle migration is influenced by factors including particle size, concentration, surface properties, and the physicochemical characteristics of the polymer matrix. Moreover, excessive exposure to certain nanomaterials may pose toxicological risks due to their potential bioaccumulation and interactions with human cells and tissues. Another notable challenge is the absence of harmonized regulations and standardized protocols for evaluating nanoparticle migration, which limits the safe and widespread industrial application of nano‐enabled food packaging systems. Consequently, detailed toxicological assessments and long‐term migration studies are essential to ensure both the safety and regulatory approval of biopolymer packaging materials containing nanofillers (Cerqueira et al. 2018; Huang et al. 2015; Souza and Fernando 2016).

**FIGURE 1:**
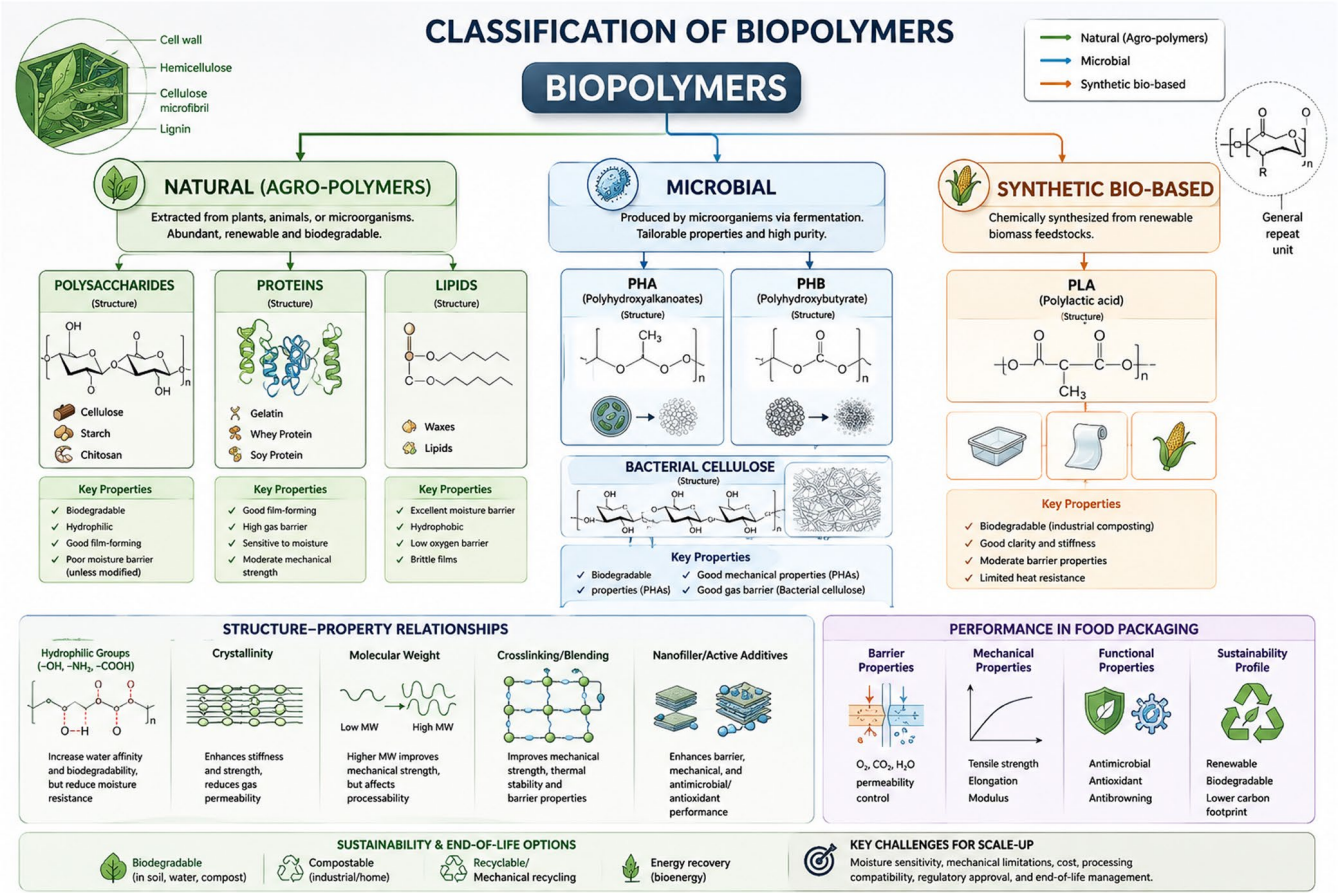
Classification of biopolymers used in food packaging according to their origin. Created with ChatGPT (OpenAI, GPT‐5.5) and edited by the authors.

Biopolymers are obtained from several natural sources including animals, plants, marine organisms, food waste, and microorganisms. Bio‐based polymers are obtained from renewable feedstocks and are generally classified into three main groups according to their origin and method of synthesis: natural biopolymers (polysaccharides, proteins, and lipids), synthetic biopolymers (including aromatic and aliphatic polymers), and microbial biopolymers produced by microorganisms (bacterial cellulose, levan, and pullulan). Based on their source, biopolymers can also be categorized as plant‐derived (cellulose, starch, and pectin) or animal‐derived (chitosan, keratin, and gelatin) (Figure 1). In addition to conventional classification, a more functional approach can be adopted to better reflect their roles in packaging technologies. From this perspective, biopolymers can be categorized as (i) barrier materials, primarily used to control gas and moisture transfer (polysaccharides), (ii) structural materials, contributing to mechanical strength and integrity (protein‐based polymers), and (iii) active materials, designed to provide additional functionalities such as antimicrobial or antioxidant activity through the incorporation of bioactive compounds. This functional classification provides a more application‐oriented framework and enables a clearer understanding of how different biopolymers contribute to packaging performance (Table 1) (Awobifa et al. 2026; Flórez et al. 2023; Oladzadabbasabadi et al. 2022). From a functional perspective, each class of biopolymers presents distinct advantages and limitations. Polysaccharide‐based materials generally exhibit excellent oxygen barrier properties and biodegradability, whereas their high hydrophilicity leads to poor moisture resistance. Protein‐based biopolymers offer good film‐forming ability and mechanical strength but may suffer from sensitivity to environmental conditions such as humidity and pH. Lipid‐based materials provide superior water vapor barrier properties, although their mechanical strength and structural integrity are often limited. Therefore, no single biopolymer class fully satisfies all packaging requirements, necessitating the development of composite and multilayer systems to balance these properties (Perera et al. 2023; Rhim et al. 2013; Rosenboom et al. 2022).

**TABLE 1:** Overview of major biopolymer classes used in food packaging.

Biopolymers	Types	Main advantages	Main limitations	Applications
Polysaccharides	Starch, cellulose, chitosan, alginate	Excellent oxygen barrier, biodegradability, film‐forming ability	Poor moisture resistance, hydrophilicity	Edible coatings, fresh produce packaging
Proteins	Gelatin, whey protein, soy protein	Good mechanical strength, gas barrier properties	Sensitivity to humidity and pH	Meat and dairy packaging
Lipids	Waxes, fatty acids	Excellent water vapor barrier	Weak mechanical properties, brittleness	Moisture‐resistant coatings
Microbial biopolymers	PHA, PHB, bacterial cellulose	Biodegradability, good gas barrier performance	High production cost, brittleness	Sustainable rigid and flexible packaging
Synthetic bio‐based polymers	PLA	Transparency, processability, compostability	Limited thermal resistance, brittleness	Beverage bottles, disposable containers

As illustrated in Figure 1, biopolymers used in food packaging can be classified according to their origin and functional characteristics, highlighting the structural diversity of materials available for green packaging applications. The figure also demonstrates how different biopolymer classes contribute distinct functional properties, including barrier performance, mechanical stability, and bioactive functionality, which are critical for the design of advanced food packaging systems.

Natural biopolymers mainly consist of polysaccharides, proteins, and lipids obtained from plants or animals. Among these, polysaccharides are among the most abundant and widely available groups of biopolymers, derived from natural resources such as plant fibers and marine organisms. Structurally, they are composed of long chains of monosaccharide units linked by glycosidic bonds (Figure 1). Due to their biodegradability, non‐allergenic nature, environmental friendliness, and cost‐effectiveness, polysaccharides have become attractive materials for surface coatings and packaging applications (Al‐Hammood et al. 2025). Their widespread availability, ease of extraction, and non‐toxic characteristics have further promoted their use in innovative food packaging systems (Li et al. 2024; Yao et al. 2024). Recent advances in coating technologies indicate that hybrid and crosslinked biopolymer coatings can significantly improve moisture resistance and mechanical stability (Oladzadabbasabadi, Manamperi, Murdoch, et al. 2026). Beyond their traditional barrier and film‐forming roles, recent studies have increasingly emphasized the multifunctional biological activities of polysaccharides, such as antioxidant, antimicrobial, and health‐promoting effects, which may further broaden their potential applications in advanced food packaging systems (Chen et al. 2022).

Polysaccharide‐based packaging materials generally demonstrate good oxygen barrier properties, which can effectively slow the respiration rate of foods, but they typically show poor resistance to water vapor due to their hydrophilic nature. They help limit the growth of bacterial and fungal contaminants within packaged products. Earlier generations of polysaccharide films typically exhibited excellent oxygen barrier properties only under dry conditions, yet recent nanocellulose‐based systems have shown prominently enhanced barrier stability under high‐humidity conditions. For instance, Zheng et al. (2026) developed a water‐resistant and transparent cellulose nanofibril film that maintained outstanding oxygen‐barrier performance even at elevated relative humidity levels (Zheng et al. 2026). However, one major drawback of these materials is their relatively poor resistance to water vapor. Commonly used polysaccharides include chitosan, starch, cellulose, pullulan, agar, alginate, and pectin. Among these, chitosan, starch, and cellulose display distinct functional characteristics that affect their suitability for packaging solutions. Chitosan is well known for its inherent antimicrobial activity and good film‐forming ability, whereas it is sensitive to moisture and exhibits limited mechanical strength. Starch‐based materials are widely available and cost‐effective, but they generally suffer from poor water vapor resistance due to their highly hydrophilic structure. In contrast, cellulose and its derivatives provide relatively better mechanical strength and structural stability, although their barrier performance against moisture remains limited. These differences highlight the importance for material selection based on specific application requirements, as well as the development of composite systems to overcome individual limitations (Long et al. 2023; Oladzadabbasabadi, Manamperi, Dekiwadia, et al. 2026; Saidi et al. 2025).

Microbial biopolymers are synthesized by bacterial or fungal strains. These polymers include polyesters such as polyhydroxybutyrate (PHB) and polyhydroxyalkanoates (PHA), as well as carbohydrate‐based polymers such as levan, pullulan, and bacterial cellulose, which are directly synthesized by microorganisms. In contrast, polylactic acid (PLA), although bio‐based, is typically produced via microbial fermentation of sugars to lactic acid followed by chemical polymerization, and therefore is not strictly classified as a microbial biopolymer. Among these materials, PHA represents an important group of microbial polyesters produced through the bacterial fermentation of sugars and lipids. PHAs are biodegradable, biocompatible, and originating from renewable resources. In terms of functional performance, PHAs exhibit tensile strength values typically ranging from 20 to 40 MPa, which are comparable to polypropylene (PP), whereas their oxygen permeability is generally lower than that of polyethylene (PE), indicating favorable gas barrier properties. However, PHAs often display higher brittleness and lower elongation at break compared to conventional plastics, which can limit their flexibility in certain packaging applications (Koller 2022; Perera et al. 2023). In general, PHA polymers exhibit good resistance to ultraviolet radiation, are insoluble in water, and show moderate resistance to hydrolytic degradation. They are soluble in chloroform and other chlorinated hydrocarbons and are considered non‐toxic. On the other hand, their resistance to acidic and alkaline environments is relatively limited. Owing to their hydrophobic nature and tunable mechanical properties, these materials are considered viable alternatives to conventional plastics, particularly due to their tunable mechanical properties and improved resistance to environmental stress under controlled conditions. As a result, microbial biopolymers have attracted increasing attention for potential applications in the food packaging industry, where they may serve as sustainable substitutes for traditional plastic materials (Koller 2022; Perera et al. 2023).

Prior to selecting a material for food packaging, its properties must be thoroughly evaluated. The selection depends on several factors including barrier performance, chemical stability, thermal behavior, mechanical strength, environmental impact, and economic feasibility (Bahramian et al. 2025). In this context, standardized testing methods are widely used to evaluate packaging performance. For example, oxygen transmission rate (OTR) and water vapor transmission rate (WVTR) are commonly determined according to ASTM D3985 and ASTM E96 standards, respectively, whereas mechanical properties such as tensile strength are measured using ASTM D882. In addition, biodegradability and compostability are assessed based on international standards such as ISO 17088 and EN 13432, which define the requirements for materials to be considered industrially compostable (He et al. 2025; Kumari et al. 2024). These characteristics are essential for maintaining food quality and enhancing preservation efficiency of packaged products. Packaging materials should also be compostable polymers, non‐toxic, and safe for direct contact with food. In addition, they should possess sufficient flexibility to bend or stretch without breaking (Li et al. 2024).

The barrier properties of biopolymers used in food packaging play a crucial role in prolonging the shelf life of packaged foods. Important barrier characteristics include resistance to gases, water vapor, organic vapors, moisture, liquids, and ultraviolet (UV) radiation, which help protect food from external environmental factors (Oladzadabbasabadi, Abedi‐Firoozjah, Tavassoli, et al. 2026). In particular, gas barrier performance is a key consideration when selecting packaging materials. Quantitatively, OTR values for biopolymer‐based films typically range from 1 to 100 cm^3^·m^−2^·day^−1^·atm^−1^ depending on composition and processing conditions, whereas conventional polyethylene materials often exhibit substantially higher OTR values (> 1000 cm^3^·m^−2^·day^−1^·atm^−1^). In contrast, WVTR values for hydrophilic biopolymers are generally high, commonly exceeding 100 g·m^−2^·day^−1^, which reflects their limited resistance to moisture compared to conventional plastics (He et al. 2025; Long et al. 2023; Perera et al. 2023). Inadequate barrier protection can lead to rapid spoilage of food products, for instance, insufficient oxygen barriers can accelerate the oxidation of fat‐rich foods (Baniasadi et al. 2026; Long et al. 2023).

Another primary purpose of packaging is to protect food products from physical damage caused by external forces such as cracking or accidental breakage. Therefore, the mechanical properties of packaging materials are essential for maintaining product integrity during storage, transportation, handling, and processing. The mechanical performance of biopolymers is largely determined by the structure of their polymer matrix. Important mechanical parameters for packaging materials include tensile, seal, hot tack, impact, and peel strengths. In quantitative terms, the tensile strength of common biopolymer‐based films such as starch, chitosan, and protein‐based materials typically ranges from 10 to 50 MPa, depending on formulation and processing conditions. For comparison, conventional plastics such as polyethylene (PE) exhibit tensile strength values of approximately 10–30 MPa, whereas polypropylene (PP) can reach 30–40 MPa. Despite comparable tensile strength in some cases, biopolymers often exhibit lower elongation at break and reduced flexibility, which may limit their performance in applications requiring high mechanical durability (He et al. 2025; Siddiqui et al. 2024). Recent findings indicate that the incorporation of bio‐based fillers and crosslinking strategies can improve tensile strength and flexibility by enhancing intermolecular interactions within the polymer matrix (Oladzadabbasabadi et al. 2024).

The optical properties of biopolymer‐based packaging materials describe how they interact with light and include characteristics such as absorbance, transmittance, color, transparency, gloss, opacity, and the ability to block UV radiation. These properties are substantial in food packaging as they influence consumer perception, product appearance, and the visibility of the packaged contents. Optical characteristics may vary depending on the composition of the material, its thickness, and the manufacturing process used (Perera et al. 2023; Rostamabadi et al. 2024).

Chemical resistance is also an important consideration because food products may contain acidic, alkaline, oily, or alcoholic components that can interact with packaging materials. Therefore, understanding the chemical characteristics of the food prior to packaging is essential. Chemical compounds may migrate into the food, cause degradation of the packaging material, or be absorbed within the biopolymer matrix, thereby altering its barrier and mechanical properties. For this reason, packaging materials must exhibit sufficient resistance to prevent the migration of harmful compounds from the packaging into food and ultimately into the human body, thereby ensuring both food safety and consumer health (Groh et al. 2021; Muncke et al. 2020).

Thermal properties reflect the behavior of a biopolymer under thermal conditions. These include parameters such as decomposition temperature, melting temperature, glass transition temperature (*T*_g_), thermal stability, and overall heat resistance. These properties are critical in food packaging as they affect the performance of materials during processing operations such as extrusion, thermoforming, and molding, as well as during storage and thermal treatments. Therefore, thermal behavior must be carefully considered during the design and development of packaging materials (Gupta et al. 2022; Hussain et al. 2024).

## 3 Packaging Technologies Based on Biopolymers

### 3.1 Active Packaging

Active packaging materials are specially designed systems developed to improve the sensory attributes of food products and preserve their quality. These systems contain functional components that actively interact with both the packaged food and the surrounding environment. Through these interactions, active packaging helps maintain food safety, protect product quality, and improve storage stability (Wang et al. 2026). In addition, such packaging provides protection from external environmental factors while regulating the internal atmosphere within the package. Active packaging systems are generally categorized into two main groups: emitters and absorbers. Emitters release certain substances, such as carbon dioxide, into the packaging environment, whereas absorbers remove unwanted compounds including oxygen, carbon dioxide, ethylene, and moisture. Unlike intelligent packaging, active packaging systems do not respond to specific external stimuli but instead function continuously to maintain optimal storage conditions (Sabaghi 2025). In general, active packaging systems are designed to modify the internal package environment through mechanisms such as oxygen scavenging, antimicrobial activity, or moisture regulation in order to preserve food quality and extend shelf life. In contrast, intelligent packaging systems primarily serve monitoring and communication functions by detecting changes in factors such as temperature, gas composition, microbial growth, or freshness indicators. Therefore, while active packaging directly interacts with the packaged food or surrounding atmosphere, intelligent packaging mainly provides information regarding product condition and storage history (Du, Sun, et al. 2023; Lou et al. 2025; Vilela et al. 2018). Several active packaging technologies have already been implemented at industrial scale in commercial food packaging applications. For example, oxygen scavenger sachets are widely used in packaged meat, bakery, and ready‐to‐eat products to reduce oxidative deterioration and preserve product quality during storage. Moisture‐absorbing pads containing silica gel or calcium oxide are commonly applied in fresh meat and seafood packaging to control excess humidity and inhibit microbial growth. In addition, antimicrobial films incorporating silver nanoparticles, chitosan, or essential oils have been investigated for commercial applications in fresh produce and dairy packaging. These examples demonstrate the growing industrial relevance of active biopolymer‐based packaging systems and their potential to improve food preservation under real storage and distribution conditions (Chan et al. 2025; Lou et al. 2025; Motelica et al. 2020).

Ethylene is a natural plant hormone that, even at low concentrations, can accelerate ripening, softening, and aging in fresh produce. This process increases the respiration rate of foods during storage and transportation, thereby shortening their shelf life. To mitigate this effect, ethylene absorbers are commonly used. These absorbers remove ethylene from the packaging environment via chemical interactions or physical adsorption inside a closed system (Vilela et al. 2018).

Carbon dioxide also contributes to extending the freshness of food by inhibiting microbial growth and reducing oxidative reactions. In contrast, the concentration of CO_2_ in packaging must be carefully controlled, as excessive levels may negatively affect product quality by altering organoleptic properties, including flavor and texture, particularly in dairy‐derived foods. Carbon dioxide absorbers can help regulate CO_2_ levels, suppress the growth of microorganisms such as yeasts, molds, and bacteria, remove excess gas, and prevent package swelling or rupture during storage (Chan et al. 2025).

Oxygen is another crucial factor affecting food stability. Its presence can initiate oxidative reactions and promote microbial spoilage, resulting in nutrient loss, discoloration, vitamin degradation, and lipid oxidation, ultimately reducing the shelf life of food products. Oxygen absorbers are therefore widely used to control oxygen levels within packaging and to limit oxygen penetration via the packaging system throughout storage. These absorbers reduce deterioration, prevent rancidity, and improve the overall barrier performance of active packaging systems (Realini and Marcos 2014; Wang et al. 2026).

**FIGURE 2:**
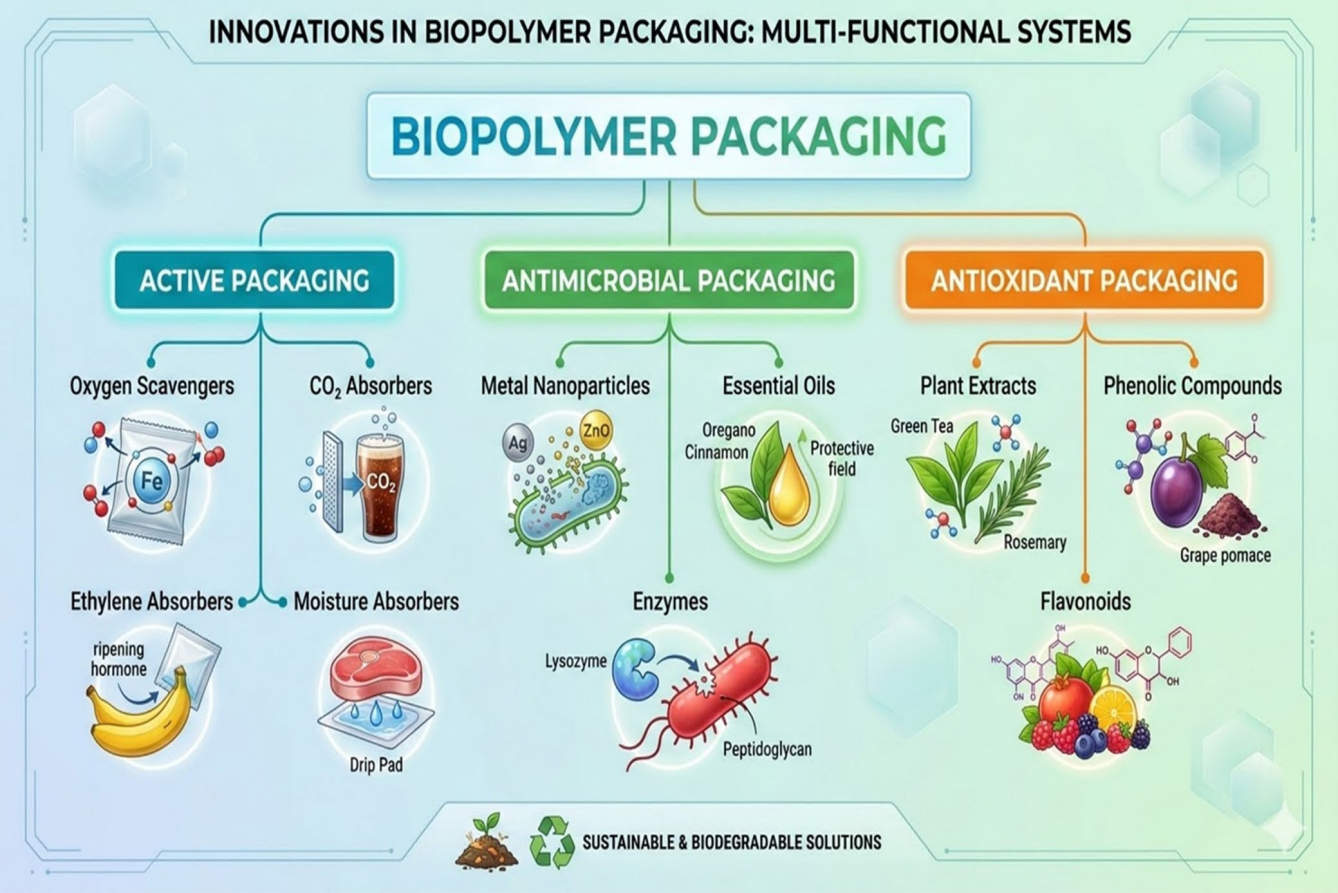
Functional categories of active biopolymer‐based food packaging systems. Created with ChatGPT (OpenAI, GPT‐5.5) and edited by the authors.

Excess moisture within sealed packages can also cause problems including microbial growth, changes in food texture, reduced transparency, and shorter shelf life. To regulate humidity levels and maintain product quality, moisture absorbers or desiccants are incorporated into packaging systems. Moisture‐adsorbing substances such as calcium oxide, silica gel, and bentonite clay are commonly used for this purpose. These materials are typically applied in the form of films, pads, or sachets placed inside the package to absorb excess moisture and protect moisture‐sensitive foods, thereby preserving their overall quality (Figure 2) (Du, Sun, et al. 2023; Kalita et al. 2026).

### 3.2 Antimicrobial Packaging

Antimicrobial packaging systems incorporate active compounds into packaging materials to inhibit or slow the growth of microorganisms. This approach contributes to reducing food waste while maintaining product quality, preserving appearance, and prolonging storage stability of food products (Rashid et al. 2026). Antimicrobial agents used in such systems can be classified into several groups: (1) organic compounds such as enzymes, organic acids, and polymers, (2) inorganic materials including metals and metal oxides, (3) essential oils such as thyme, clove, and oregano, (4) plant extracts derived from sources like rosemary, green tea, ginger, and garlic, (5) bioactive compounds such as thymol and carvacrol, and (6) antimicrobial peptides including lactoferrin and nisin (Motelica et al. 2020; Nandhini et al. 2024).

Metal nanoparticles such as zinc, silver, and copper are widely incorporated into bio‐nanocomposite materials for antimicrobial applications in the food industry. Chitosan‐based biopolymers are also commonly used due to their inherent antimicrobial properties. Films developed from chitosan demonstrate enhanced antimicrobial activity, effectively suppress microbial growth and prolong the shelf life of packaged foods. Consequently, chitosan‐based films are considered a promising and novel approach for active food packaging technologies (Figure 2) (Jiang et al. 2023; Porta et al. 2022).

Although active packaging systems demonstrate notable antimicrobial potential, safety and regulatory issues continue to represent major barriers to their large‐scale industrial implementation. Antimicrobial compounds embedded within packaging materials can migrate into food during storage, especially when exposed to elevated temperatures, extended contact periods or foods with high fat or acid content. The uncontrolled migration of certain substances, particularly metal nanoparticles such as silver and zinc oxide, may pose toxicological risks due to their possible accumulation in living organisms and associated adverse health effects. Therefore, the migration characteristics and toxicological profiles of antimicrobial packaging materials must be thoroughly assessed prior to commercial use. Furthermore, regulatory authorities including the U.S. Food and Drug Administration and the European Food Safety Authority enforce stringent regulations governing antimicrobial agents used in food‐contact materials, covering aspects such as migration thresholds, safety testing, and risk assessment protocols. Consequently, standardized migration analyses together with comprehensive toxicological evaluations are essential to guarantee the safe utilization of antimicrobial biopolymer‐based packaging systems (Geueke et al. 2018; Groh et al. 2021; Muncke et al. 2020).

### 3.3 Antioxidant Packaging

Lipid oxidation and microbial growth are two major factors that adversely affect the shelf life and quality of food products. Consequently, the food industry focuses on delaying oxidative processes to prevent lipid degradation, texture deterioration, off‐flavor development, and color deterioration. Traditionally, synthetic antioxidants have been widely used to maintain food quality. Conversely, rising concerns over the possible health risks associated with synthetic antioxidants have prompted the exploration of natural antioxidant alternatives for food packaging applications (Bruni et al. 2025; Munekata et al. 2020).

Synthetic antioxidants such as butylated hydroxytoluene (BHT) and butylated hydroxyanisole (BHA) are widely utilized in food preservation because of their high oxidative stability, strong antioxidant performance, and relatively low cost. However, growing concerns about their possible toxicological risks and long‐term health effects have increased interest in the development of natural antioxidant alternatives. Natural antioxidants derived from plant extracts, phenolic compounds, and essential oils are generally regarded as safer and more eco‐friendly, whereas also providing additional functional benefits including antimicrobial activity. Despite these advantages, natural antioxidants commonly show lower thermal stability, greater sensitivity to environmental factors, and more variable antioxidant effectiveness than synthetic antioxidants. Consequently, improving the stability, controlled‐release characteristics, and compatibility of natural antioxidants within biopolymer‐based matrices remains a major research priority in the development of active food packaging systems (Bruni et al. 2025; Khan et al. 2023; Munekata et al. 2020).

Natural antioxidants contain several categories of compounds such as (1) plant extracts derived from sources like mint, oregano, rosemary, and murta, (2) phenolic compounds including caffeic acid phenethyl ester and curcumin, (3) essential oils containing bergamot, cinnamon, and lemongrass, and (4) flavonoids such as quercetin and catechin. These natural substances are increasingly incorporated into packaging materials to enhance their antioxidant functionality. For example, cellulose‐based films containing ascorbyl dipalmitate nanoparticles and curcumin have demonstrated effective antioxidant activity in packaging systems. In a similar manner, gelatin‐based films enriched with chitosan nanoparticles and tea polyphenols exhibit enhanced antioxidant properties, making them promising candidates for use in active food packaging applications (Figure 2) (Khan et al. 2023; Perera et al. 2023). Recent applications demonstrate improved shelf‐life extension in perishable foods through the use of active biopolymer‐based packaging systems (Elewi et al. 2025).

## 4 The Use of Biopolymers in Food Packaging Applications

**FIGURE 3:**
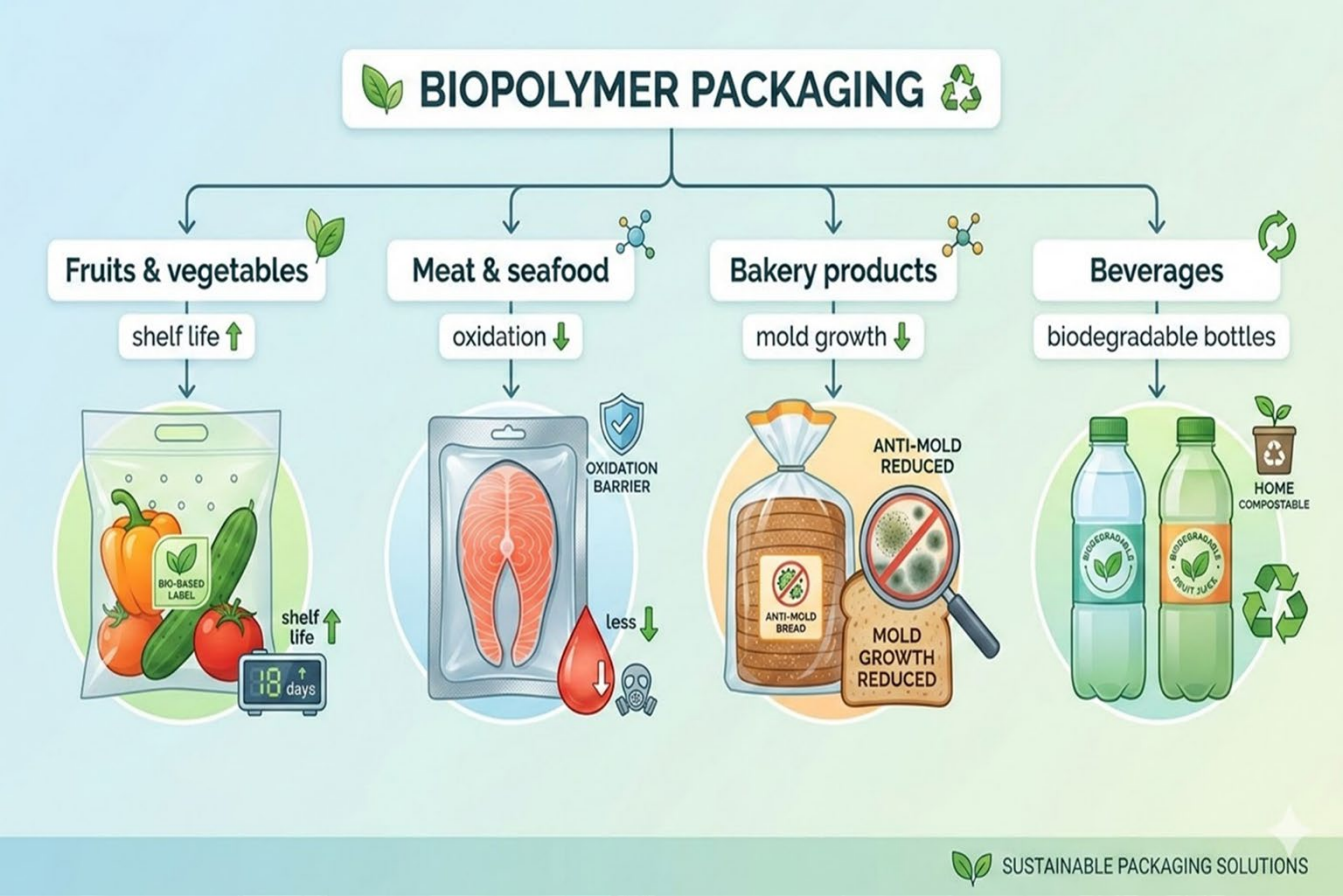
The use of biopolymers in food packaging applications. Created with ChatGPT (OpenAI, GPT‐5.5) and edited by the authors.

The accelerating issue of global pollution has heightened consciousness about the environmental impact associated with plastic waste, underscoring the necessity for sustainable alternatives to traditional plastic packaging. In response to the increasing demand for reliable, natural, and environmentally friendly solutions, food preservation technologies continue to evolve to maintain product quality while decreasing environmental burdens. These strategies focus on preserving the nutritional content and sensory properties of food while reducing adverse environmental effects (Geyer et al. 2017). In this regard, biopolymers have emerged as promising alternatives that can boost the performance and efficiency of food packaging materials. The integration of bioactive compounds into packaging systems has attracted great interest due to their accessibility and cost‐effectiveness, and their effectiveness as functional additives that improve the properties of biopolymer‐based materials (Abraham et al. 2025). These eco‐friendly materials provide a natural and sustainable approach to enhancing food preservation without compromising food safety or exacerbating the issue of plastic waste (Figure 3) (Ramli 2026).

As summarized in Table 2, numerous studies have demonstrated that biopolymer‐based coatings significantly extend the shelf life of fresh fruits and vegetables by reducing moisture loss, inhibiting microbial growth, and delaying ripening. Various biopolymer coatings, particularly those based on chitosan, starch, and alginate, have been successfully applied to fruits such as bananas, apples, and strawberries (Abraham et al. 2025). Moreover, incorporating natural bioactive compounds, including flavonoids, plant extracts, and essential oils, further enhances the antimicrobial and antioxidant properties of these coatings, thereby improving food quality and delaying product deterioration during storage (Du, Shi, et al. 2023; Fauzi et al. 2025; Hossain and Iqbal 2016; Hu et al. 2022; Ramli et al. 2024; Singh and Packirisamy 2022).

**TABLE 2:** Recent applications of biopolymer‐based edible coatings and active packaging systems in food preservation.

Biopolymer/coating material	Active compound	Food product	Main findings	Critical evaluation (effectiveness/limitations/scalability)	References
Chitosan coating	—	Banana	Reduced weight loss and delayed color change, extending banana shelf life	An effective and low‐cost coating strategy for delaying ripening. However, limited moisture resistance and relatively weak mechanical stability may limit long‐term industrial applications	Hossain and Iqbal (2016)
Chitosan film	Persimmon peel extract (phenolics & flavonoids)	Banana	Enhanced antioxidant and barrier properties, slowing banana ripening and quality loss	Natural phenolic incorporation improved antioxidant activity, although variability in plant extract composition may affect reproducibility and consistency during scale‐up	Hu et al. (2022)
Chitosan/cellulose nanocrystal composite coating	—	Banana	Improved mechanical strength and antimicrobial activity, extending the shelf life	Nanocellulose reinforcement improved film performance, but large‐scale nanocomposite fabrication and homogeneous filler dispersion remain challenging	Du, Shi, et al. (2023)
Silk fibroin nanofiber coating	Nano‐curcumin	Banana	Reduced weight loss and extended the shelf life	Nano‐curcumin improved preservation efficiency, yet electrospinning‐based fabrication may increase processing complexity and production cost	Singh and Packirisamy (2022)
Chitosan nanoparticle coating	*Aloe vera* or Moringa extract	Banana	Prolonged storage by reducing microbial growth and water loss	Plant extracts boosted antimicrobial activity, although nanoparticle stability and possible sensory alterations require further investigation before commercialization	Ramli et al. (2024)
ZnO‐alginate beads with immobilized black radish peroxidase	Black radish peroxidase + ZnO nanoparticles	Mushroom	Improved antimicrobial, antioxidant, and catalytic properties with enhanced multifunctionality	Highly effective multifunctional system, but potential nanoparticle migration, enzyme stability during long‐term storage, and industrial‐scale immobilization costs remain important limitations	Bayrak (2025)
EPS‐modified alginate films	Exopolysaccharides (EPS)	Fresh‐cut apples	Improved barrier, antioxidant, antimicrobial, and antibrowning properties	Promising biodegradable alternative with improved film performance, although moisture sensitivity and scale‐up consistency of EPS production may limit industrial implementation	Bayrak and Omeroglu (2026)
Chitosan nanoparticles	Turmeric oil	*Agaricus bisporus* (mushroom)	Controlled release system prolonged shelf life and preserved postharvest quality	Controlled‐release functionality improved preservation efficiency. However, the complexity of nanoparticle fabrication and potential sensory impacts of essential oils may limit commercialization	Pleșoianu and Nour (2022)
Soy protein–cysteine edible coating with MAP	Cysteine	Fresh‐cut eggplant	Delayed browning and maintained physicochemical quality during storage	Effective antibrowning approach for minimally processed vegetables, although protein‐based coatings may exhibit lower water vapor resistance and reduced mechanical stability under high humidity conditions	Ghidelli et al. (2014)
Polysaccharide‐based edible coatings	Natural polysaccharides	Eggplant	Improved postharvest quality and delayed deterioration	Environmentally friendly and cost‐effective systems, but their hydrophilic nature generally limits moisture‐barrier performance compared with synthetic plastics	Gonzales and Benitez (2019)
Rice starch edible films	Curcumin nanoparticles	Strawberry	Antimicrobial activity and improved preservation during storage	The incorporation of curcumin nanoparticles enhanced antimicrobial activity, whereas starch‐based matrices still suffer from limited mechanical strength and high water sensitivity	Wang et al. (2025)

Comparative analysis of recent studies indicates that multifunctional biopolymer systems incorporating nanoparticles, immobilized enzymes, or controlled‐release bioactive compounds generally exhibit superior preservation performance compared with conventional single‐component edible coatings. For example, ZnO‐alginate systems containing immobilized black radish peroxidase provided simultaneous antimicrobial, antioxidant, and catalytic activities, whereas chitosan nanoparticle systems loaded with turmeric oil demonstrated effective controlled‐release functionality and improved postharvest preservation of mushrooms. In contrast, simpler polysaccharide or protein‐based coatings are generally more cost‐effective and easier to scale up industrially. Nonetheless, they often display weaker moisture‐barrier properties and lower mechanical stability under high‐humidity conditions (Bayrak 2025; Bayrak and Omeroglu 2026; Ghidelli et al. 2014; Gonzales and Benitez 2019; Valizadeh et al. 2021; Wang et al. 2025). Despite the promising effectiveness of advanced multifunctional systems, several important limitations still hinder their large‐scale industrial implementation. Nanoparticle‐containing coatings may raise concerns regarding migration behavior, regulatory approval, and long‐term safety. In addition, immobilized enzyme systems and nano‐enabled coatings often require more complex fabrication methods and incur higher production costs than conventional edible coatings. Therefore, future research should focus not only on improving preservation efficiency but also on enhancing scalability, economic feasibility, long‐term stability, and industrial applicability of biopolymer‐based food packaging systems.

### 4.1 Packaging for Fruits and Vegetables

Fruits and vegetables are highly perishable products and are therefore vulnerable to rapid quality deterioration after harvest. This deterioration is influenced by several factors, including postharvest handling practices, processing methods, and environmental conditions such as temperature, moisture, humidity, and exposure to sunlight. To overcome these limitations, there is a growing need for advanced technologies which can enhance production and delivery systems while reducing quality losses and improving storage stability of fresh produce. Following harvest, fruits generally have a limited shelf life due to microbial activity, high respiration rates, and moisture loss (Venkatesan and Muniyan 2024). Controlling environmental factors such as temperature, moisture, light, and the levels of gases including carbon dioxide, oxygen, and ethylene can help slow respiration and transpiration processes, thus maintaining the storage life of fruits and vegetables. Numerous studies have highlighted the effectiveness of biopolymer‐based edible coatings in extending the postharvest shelf life of fresh produce. In particular, chitosan‐, alginate‐, and starch‐based coatings enriched with natural antimicrobial or antioxidant agents have been widely investigated (Pillai et al. 2024; Saberi and Golding 2018). These coatings form semi‐permeable barriers that reduce moisture loss, regulate gas exchange, and inhibit microbial growth, thereby delaying ripening and senescence processes in fruits and vegetables (Rajapakshe et al. 2026; Rizzo 2025).

### 4.2 Packaging for Meat, Poultry, and Seafood

Meat, fish, and other animal‐derived products are highly perishable due to their biological composition and can deteriorate rapidly if not stored under appropriate conditions. Inadequate storage may lead to undesirable changes in flavor, texture, oxidation, and appearance, which can ultimately result in spoilage and potential food safety risks. Therefore, proper packaging of meat products, whether fresh, frozen, or processed, as well as fish and other animal‐based foods is essential to prevent deterioration and prolong their shelf life (McMillin 2017; Zouharová et al. 2023). Meat products, including both fresh and processed varieties, are important sources of dietary protein for a large portion of the global population. In this regard, edible films and coatings have emerged as an innovative strategy for the preservation and packaging of these products. Such coatings and films function as protective barriers that limit microbial growth, reduce moisture loss, prevent the accumulation of spoilage‐related compounds, and inhibit the oxidation of proteins, lipids, and pigments. Consequently, these packaging systems maintain freshness and sensory quality of the products (Kumar et al. 2022; Uysal‐Unalan et al. 2024).

### 4.3 Packaging for Bakery, Confectionery and Beverage Products

Bakery and confectionery products such as bread, cookies, cakes, pastries, candies, and other sweets are highly susceptible to contamination by yeasts and molds, which can lead to unpleasant odors, off‐flavors, and visible defects. The growth of these microorganisms can compromise both the safety and the sensory quality of such products. To handle this concern, edible films and coatings are commonly applied through techniques such as wrapping, dipping, or spraying. These coating materials, often incorporated with antimicrobial or antioxidant agents, create a protective layer that limits microbial growth and contributes to prolonging storage stability of bakery and confectionery items (Qian et al. 2021; Upadhyay et al. 2024).

In recent years, biopolymers have also gained increasing attention in beverage packaging as greener alternatives to traditional petroleum‐based plastics. Their biodegradability, non‐toxic nature, and ability to form films and coatings make them suitable for packaging a wide range of beverages including water, juices, milk, and carbonated drinks. Among these materials, polylactic acid (PLA), derived from renewable sources such as corn starch or sugarcane, is one of the most widely studied biopolymers for beverage packaging. Its transparency, ease of processing, and compostability make it particularly suitable for manufacturing bottles used for cold beverages such as water and fruit juices (Banerjee et al. 2025; Ncube et al. 2021).

## 5 Environmental Benefits of Biopolymer‐Based Packaging

**FIGURE 4:**
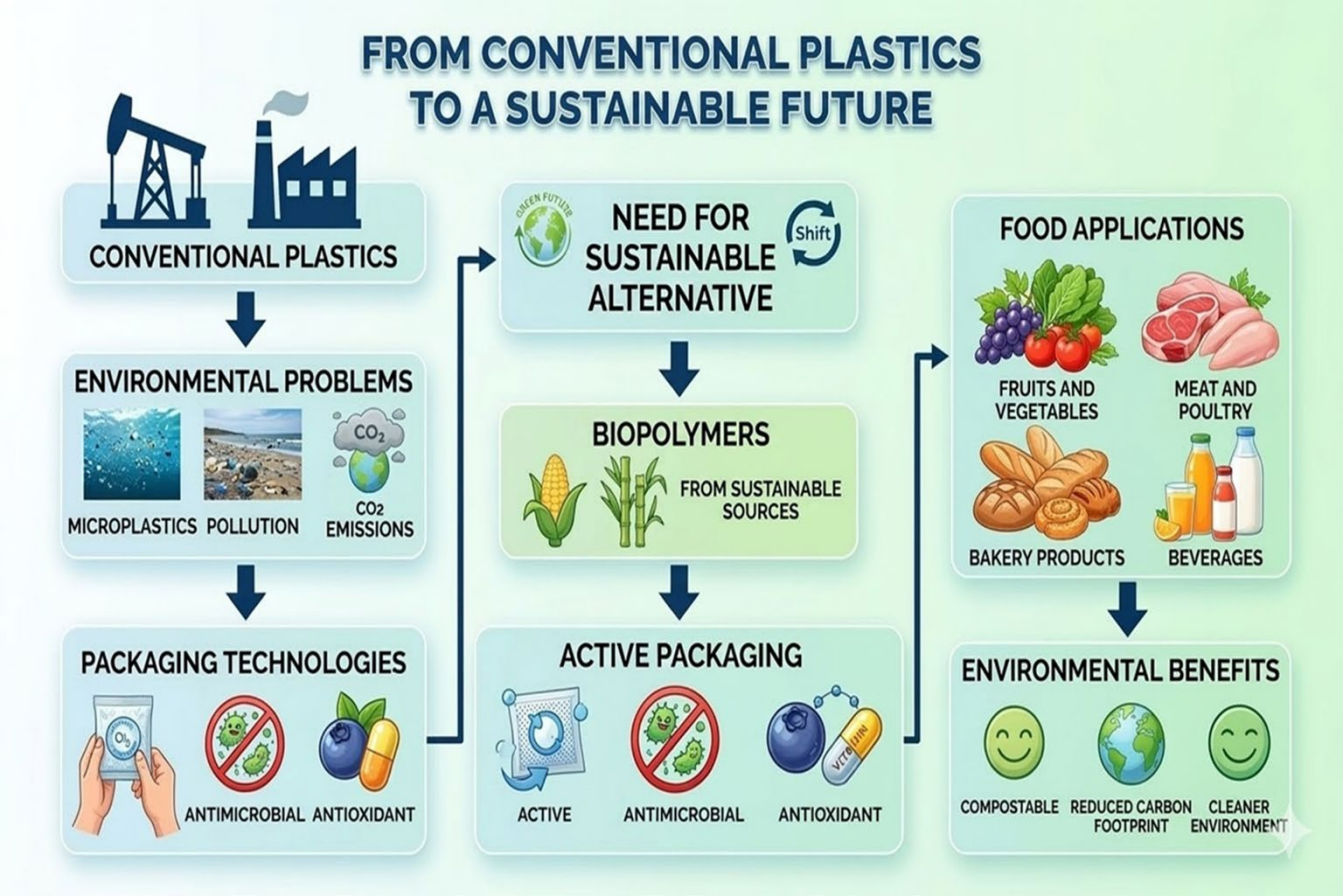
Comparison of conventional plastics and biopolymer‐based packaging materials. Created with ChatGPT (OpenAI, GPT‐5.5) and edited by the authors.

Plastics, particularly single‐use plastics, continue to dominate the food packaging sector. In contrast, the non‐biodegradable nature of conventional plastics represents a major environmental concern and has become one of the primary reasons for exploring alternative packaging materials such as biopolymers. Transitioning gradually to bio‐based packaging can reduce dependence on conventional plastic materials and lessen the environmental burden associated with plastic waste disposal (Ncube et al. 2021; Rosenboom et al. 2022). Although biopolymers have been widely promoted for food packaging applications, they generally exhibit inferior mechanical strength and weaker barrier properties compared with traditional plastics. In addition, many biopolymers are highly sensitive to moisture, which can lead to rapid degradation of films. Because of these limitations, some biopolymers are not naturally suitable for food packaging, as their structural and chemical characteristics may compromise the shelf life of packaged foods. Consequently, conventional plastic materials are still frequently preferred in many packaging applications (Figure 4) (Banu and Sharmila 2023; Gupta et al. 2024).

To address these limitations, biopolymers intended for food packaging often require reinforcement with specific additives or fillers. Such modifications can significantly enhance desirable properties including antimicrobial activity, barrier performance, and thermal stability. Despite these challenges, biopolymers offer notable environmental advantages over conventional plastics, particularly because their production and disposal typically result in lower greenhouse gas emissions. Nevertheless, the complex structure of biopolymers can also create challenges related to processing, treatment, and recycling (Dilkes‐Hoffman et al. 2019; Sapna et al. 2024). An important feature of many biopolymer‐based packaging materials is their compostability. Compostable packaging materials, such as those derived from corn starch, can substantially reduce landfill waste (Figure 4). As these materials decompose, the nutrients they release can return to the soil, contributing to the development of a circular economy through efficient resource reuse and reduced environmental impact. Additionally, biopolymer‐based packaging can contribute to reducing food waste. For instance, active packaging systems made from biopolymers can reduce spoilage during storage, thereby decreasing the amount of food discarded and ultimately reducing the volume of waste sent to landfills (Figure 2).

Life‐cycle assessment (LCA) has become a prominent tool for evaluating the overall environmental performance of biopolymer‐based food packaging materials. Unlike conventional assessments focused only on biodegradability, LCA considers the entire life cycle of packaging systems, including raw material extraction, agricultural production, polymer processing, transportation, usage, and end‐of‐life management such as recycling, composting, or landfill disposal. Several studies have reported that biopolymer‐based packaging materials can reduce greenhouse gas emissions and fossil resource consumption compared with petroleum‐based plastics, particularly when renewable feedstocks and industrial composting systems are utilized. On the other hand, the environmental benefits of biopolymers are not always absolute, as some production processes may involve high energy consumption, intensive agricultural resource use, or water demand. In addition, variations in composting infrastructure, waste management systems, and regional recycling capabilities can substantially influence LCA outcomes. Therefore, standardized and region‐specific LCA studies are necessary to accurately evaluate the true environmental sustainability of biopolymer‐based packaging systems and to avoid misleading assumptions regarding their ecological advantages (Dilkes‐Hoffman et al. 2018; Geueke et al. 2018; Kakadellis and Harris 2020).

**FIGURE 5:**
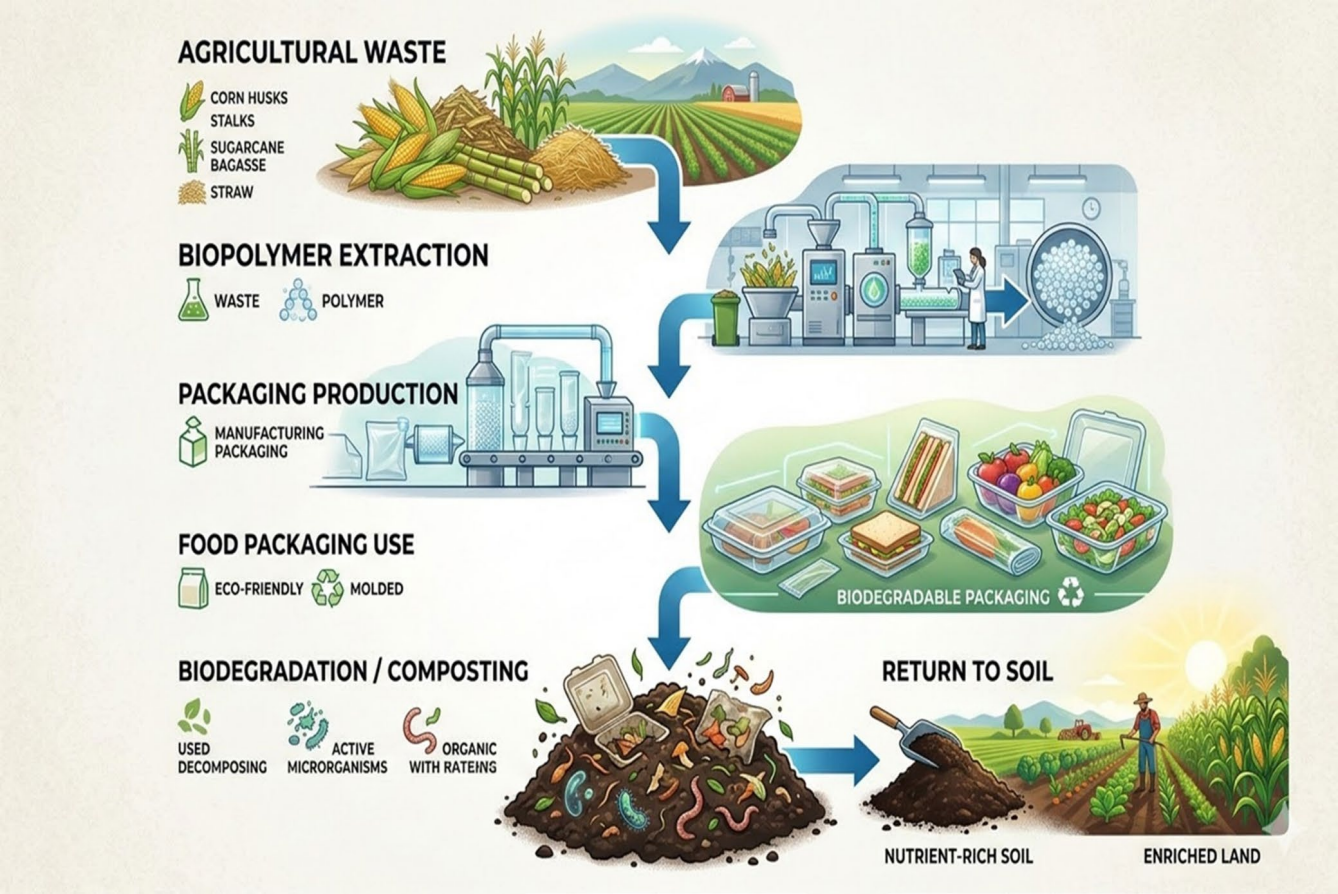
Environmental benefits of biopolymer‐based packaging. Created with ChatGPT (OpenAI, GPT‐5.5) and edited by the authors.

The food packaging industry is undergoing a significant transformation as it increasingly adopts circular economy principles, particularly through the valorization of waste generated by agro‐food industries. Converting these by‐products into materials for food packaging films has emerged as an innovative and rapidly growing strategy. This approach not only creates value‐added materials with commercial potential but also helps reduce the environmental burden associated with the disposal of agro‐based waste (Figure 5) (Ahmed et al. 2025; Czerwinski et al. 2021).

A key advantage of bio‐based packaging lies in the development of edible packaging materials. Edible films can be directly applied to food products, enabling consumers to consume the packaging together with the food. This practice can substantially reduce post‐consumer waste. Moreover, it offers several benefits, including decreasing dependence on conventional plastic packaging, providing consumers with an additional edible component, and lowering the overall amount of waste produced (Kumar et al. 2022; Sapper and Chiralt 2018). Even when these packaging materials are not consumed, they remain environmentally friendly due to their biodegradable nature, ensuring that they do not contribute to long‐term environmental pollution. Such solutions strongly support the concept of a circular economy by promoting a circular waste management approach. As a result, these technologies are gaining increasing interest within the food and beverage industries. In addition to their environmental benefits, edible films also correspond with evolving consumer preferences and expectations, whereas aligning with regulatory efforts aimed at promoting more eco‐friendly packaging alternatives (Arshad et al. 2025; Petkoska et al. 2021; Ribeiro et al. 2024).

Although food safety regulations vary across countries, the core principles governing them are generally consistent. Because food packaging safety is a critical concern, strict regulatory standards established by relevant authorities must be followed. Packaging materials are required to comply with Generally Recognized as Safe (GRAS) criteria and Good Manufacturing Practices (GMP) in accordance with regulations set by authorities such as the U.S. Food and Drug Administration (FDA). Regulatory frameworks differ across regions. In the United States, the FDA evaluates food contact materials primarily based on the GRAS status and premarket approval processes. In contrast, the European Food Safety Authority (EFSA) applies a more precautionary approach, requiring comprehensive risk assessments, migration testing, and strict compliance with specific regulations such as EU Regulation No. 10/2011 for plastic food contact materials. These differences highlight the need for harmonization of international regulations to facilitate the global commercialization of biopolymer‐based packaging materials (Table 3) (Geueke et al. 2018; Sapna et al. 2025).

**TABLE 3:** Major regulatory frameworks relevant to biopolymer‐based food packaging materials.

Regulatory	Region	Main focus	Key requirements
FDA (Food and Drug Administration)	United States	Food‐contact material safety	GRAS status, migration testing, premarket approval
EFSA (European Food Safety Authority)	European Union	Risk assessment of food‐contact materials	Migration limits, toxicological evaluation, compliance with EU Regulation No. 10/2011
GMP (Good Manufacturing Practices)	International	Manufacturing and processing safety	Hygienic production, traceability, quality assurance
EN 13432	European Union	Compostability standards	Biodegradability, disintegration, ecotoxicity assessment
ISO 17088	International	Compostable plastics specifications	Compostability and environmental compatibility
Migration testing regulations	Multiple regions	Food‐contact safety	Assessment of chemical migration into food matrices

Toxicological and allergenicity assessments are also essential, particularly for packaging materials that incorporate antimicrobial agents such as essential oils. Even if these additives enhance functionality, materials that do not meet safety requirements cannot be approved for use in food applications. Approval procedures and regulatory requirements may differ depending on national regulations or the specific standards required for export to certain countries. Proper labeling is another important regulatory requirement. Ingredient disclosure must be clearly provided, and information regarding potential allergens such as those present in coatings or film formulations must be included (Alkan and Pauliuk 2025; Arifin et al. 2023). Edible films and coatings are generally classified as food ingredients and therefore must possess GRAS status. Furthermore, certain modifications introduced during the development of edible films, such as the use of cross‐linking agents, may present potential health risks and must be carefully evaluated to ensure consumer safety (He et al. 2024).

Food packaging constitutes a large and continuously expanding market, driven by the need to overcome limitations of conventional packaging systems. Increasing awareness of environmental sustainability and public health has stimulated research into improved packaging alternatives. This shift has significantly increased interest in biopolymers for food packaging while also prompting the introduction of supportive legislation and regulatory frameworks (Ranade et al. 2025; Stoica et al. 2024). To mitigate the environmental impact of conventional plastics, many countries have implemented policies such as taxes and bans on single‐use plastics, particularly in food packaging applications. Recycling plastic packaging is another strategy that helps reduce environmental pollution (Geyer et al. 2017). However, recycling biopolymeric packaging presents certain challenges. In addition to regulatory and sorting challenges, the recycling of biopolymer‐based packaging materials also faces several technical limitations. One major issue is the incompatibility between different biopolymer matrices or between biopolymers and conventional petroleum‐based plastics during mechanical recycling processes. Such incompatibilities may result in phase separation, poor interfacial adhesion, and deterioration of mechanical performance in recycled materials. Furthermore, repeated thermal processing during extrusion or re‐melting can induce hydrolytic and thermo‐oxidative degradation of biopolymers such as PLA and starch‐based materials, leading to reductions in molecular weight, tensile strength, and barrier properties. The high sensitivity of many biopolymers to moisture and temperature further complicates recycling operations and may reduce processing stability during industrial‐scale reprocessing. Therefore, improving recycling compatibility and developing efficient separation and stabilization strategies remain important priorities for the sustainable implementation of biopolymer‐based packaging systems (La Mantia et al. 2020; Niaounakis 2019). Biopolymers are frequently combined with other plastic materials, which complicates their collection, sorting, and subsequent recycling processes. Therefore, regulatory requirements related to the recycling of packaging biopolymers must be carefully addressed. These include ensuring traceability, preventing misuse, and separating food‐contact materials from non‐food‐contact materials during recycling processes (Muncke et al. 2020; Rosenboom et al. 2022). Another important regulatory consideration is the inertness of packaging materials, as any unwanted interaction between packaging and food may compromise food quality or pose potential health risks. Although traceability is an essential regulatory parameter, maintaining it can be particularly challenging in post‐consumer scenarios. In addition, the potential presence of non‐intentionally added substances must also be evaluated. Overall, the establishment of appropriate regulations and compliance with these standards plays a vital role in supporting and promoting the use of biopolymers in food packaging (Franz and Welle 2022; Groh et al. 2019; Miralles et al. 2025).

## 6 Challenges and Future Perspectives

Despite substantial advances in the development of biopolymer‐based food packaging materials, several challenges remain that hinder their large‐scale industrial implementation. Recent studies indicate that significant research efforts have focused on improving the functional properties of biopolymer‐based packaging materials through nanotechnology and polymer blending strategies (Bayrak 2025). For instance, several studies have demonstrated that incorporating nanofillers such as zinc oxide, silver nanoparticles, or nanocellulose can substantially enhance the mechanical strength, barrier properties, and antimicrobial activity of biodegradable films (Bayrak and Ozdemir 2025; Wawrzyńczak et al. 2024). Similarly, the development of multilayer biopolymer structures has been proposed as an effective strategy to overcome the inherent limitations of single‐layer biodegradable films (Jamróz et al. 2019). These multilayer systems combine different polymers with complementary properties, thereby improving moisture resistance, oxygen barrier performance, and overall stability of packaging materials. Such advancements highlight the growing interest in designing multifunctional biopolymer‐based packaging systems that can compete with conventional petroleum‐based plastics.

Despite substantial progress in the development of biopolymer‐based packaging materials, their extensive industrial implementation remains challenging. Key barriers include high production costs, limited availability of raw materials at industrial scale, and incompatibility with existing large‐scale processing technologies such as extrusion and injection molding. In addition, maintaining consistent material properties during scale‐up is often difficult due to variations in raw material composition and processing conditions. Compared to conventional plastics, which benefit from well‐established and cost‐efficient manufacturing infrastructures, biopolymer‐based materials often require additional modification or blending to meet industrial performance standards. Addressing these challenges requires the development of cost‐effective production methods, improved processing technologies, and stronger integration with existing industrial systems (Perera et al. 2023; Rosenboom et al. 2022; Sharma et al. 2025).

Future research should prioritize enhancing the barrier and mechanical performance of biopolymers through advanced material engineering approaches such as polymer blending, multilayer film design, and the incorporation of functional nanofillers. In particular, the development of bio‐nanocomposite systems has demonstrated significant promise in improving the functional properties of biodegradable packaging materials while preserving their environmental compatibility.

**FIGURE 6:**
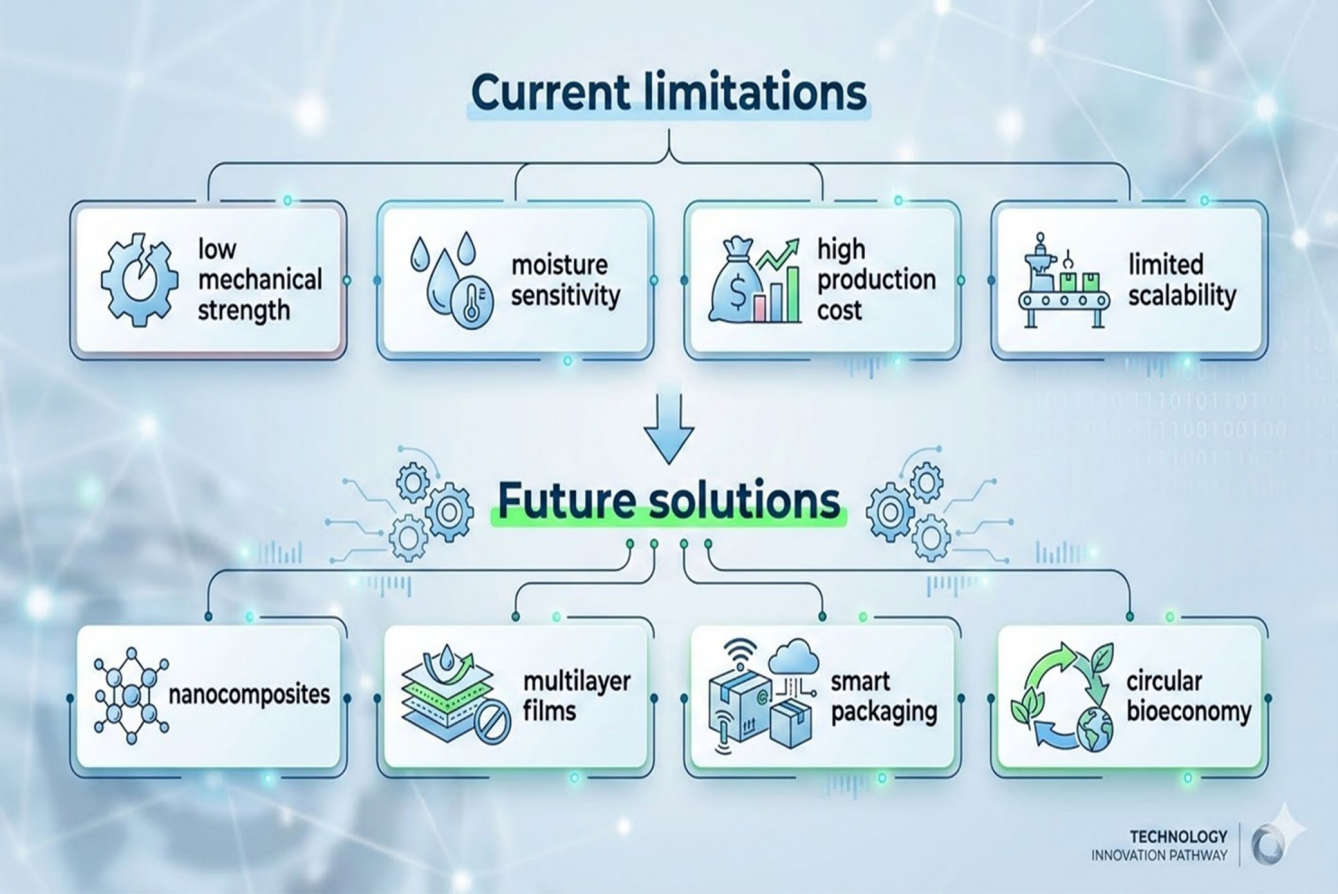
Current challenges and future research directions for biopolymer‐based food packaging. Created with ChatGPT (OpenAI, GPT‐5.5) and edited by the authors.

Although substantial advances have been achieved in the development of biopolymer‐based food packaging materials, several critical research gaps still require further investigation. Most existing studies are primarily conducted at the laboratory scale, whereas the long‐term performance, scalability, and processing stability of these materials under industrial manufacturing conditions remain insufficiently explored. Moreover, despite the widespread characterization of biopolymers as biodegradable, comprehensive evaluations of their degradation behavior in real environmental settings, such as soil and marine ecosystems, are still scarce. Another important limitation is the absence of standardized protocols for assessing multifunctional active packaging systems, particularly those containing antimicrobial nanoparticles or bioactive agents. In addition, the long‐term interactions between biopolymer matrices and active additives remain inadequately understood, especially in terms of migration behavior, physicochemical stability, and potential food safety concerns. Furthermore, additional techno‐economic evaluations and LCA studies are necessary to determine the industrial feasibility and market competitiveness of advanced biopolymer‐based packaging systems compared with conventional petroleum‐derived plastics (Figure 6).

Furthermore, the integration of intelligent and smart packaging technologies into biopolymer matrices represents a rapidly emerging area of research. Smart packaging systems capable of monitoring food freshness, detecting spoilage‐related indicators, or responding to environmental stimuli have the potential to significantly improve food safety and minimize food waste across the supply chain. In parallel, progress in biotechnology and green chemistry is expected to enable the production of innovative bio‐based polymers derived from renewable feedstocks, including agricultural by‐products, food processing waste, and microbial fermentation, thereby supporting the development of circular bioeconomy models.

Another critical research priority involves conducting comprehensive LCA to systematically evaluate the environmental impacts of biopolymer‐based packaging in comparison with conventional petroleum‐based plastics. Such analyses are essential to ensure that newly developed materials deliver genuine sustainability advantages throughout their entire life cycle. Finally, stronger collaboration among academic researchers, industry stakeholders, and regulatory bodies will be crucial for overcoming current technical, economic, and regulatory challenges and for accelerating the global transition toward sustainable food packaging solutions.

## 7 Conclusion

Biopolymer‐based food packaging materials have gained considerable attention as sustainable alternatives to conventional petroleum‐derived plastics because of their biodegradability, renewable sourcing, and potential contribution to environmentally responsible food systems. Recent progress in polymer engineering, nanocomposite development, and active packaging technologies has substantially boosted their functional properties including antimicrobial effectiveness, antioxidant performance, and barrier characteristics. Nevertheless, several major challenges continue to hinder their widespread industrial adoption, such as elevated production costs, sensitivity to moisture, limited thermal and processing stability, incompatibility during recycling processes, and the lack of sufficient long‐term performance evaluations under realistic storage and environmental conditions.

Future advancements in this area will require not only continued material innovation but also the establishment of standardized characterization and testing protocols, detailed toxicological and migration studies, and robust LCA frameworks. Particular attention should be directed toward the development of scalable production methods, efficient composting and recycling systems, and the incorporation of intelligent packaging features into biodegradable materials. Furthermore, strengthened collaboration among researchers, industrial stakeholders, and regulatory agencies will play a critical role in accelerating the commercialization and broader adoption of safe, high‐performance, and environmentally sustainable biopolymer‐based food packaging solutions.

## Author Contributions

**Songul Bayrak:** conceptualization, investigation, writing – original draft. **Mehmet Akif Omeroglu:** conceptualization, investigation, writing – original draft, writing – review and editing.

## Funding

The authors have nothing to report.

## Conflicts of Interest

The authors declare no conflicts of interest.

## Data Availability

The data that support the findings of this study are available from the corresponding author upon reasonable request.
